# Hybrid Electrodes by *In-Situ* Integration of Graphene and Carbon-Nanotubes in Polypyrrole for Supercapacitors

**DOI:** 10.1038/srep14445

**Published:** 2015-09-23

**Authors:** Ashish Aphale, Krushangi Maisuria, Manoj K. Mahapatra, Angela Santiago, Prabhakar Singh, Prabir Patra

**Affiliations:** 1 Department of Biomedical Engineering, University of Bridgeport, Bridgeport, CT-06604; 2 Fairfield Ludlowe High School, Fairfield, CT-06824; 3 Department of Materials Science and Engineering, Center for Clean Energy Engineering, University of Connecticut, Storrs, CT-06269; 4 Department of Chemistry, University of Bridgeport, Bridgeport, CT-06604; 5 Department of Mechanical Engineering, University of Bridgeport, Bridgeport, CT-06604.

## Abstract

Supercapacitors also known as electrochemical capacitors, that store energy via either Faradaic or non-Faradaic processes, have recently grown popularity mainly because they complement, and can even replace, conventional energy storage systems in variety of applications. Supercapacitor performance can be improved significantly by developing new nanocomposite electrodes which utilizes both the energy storage processes simultaneously. Here we report, fabrication of the freestanding hybrid electrodes, by incorporating graphene and carbon nanotubes (CNT) in pyrrole monomer via its *in-situ* polymerization. At the scan rate of 5 mV s^−1^, the specific capacitance of the polypyrrole-CNT-graphene (PCG) electrode film was 453 F g^−1^ with ultrahigh energy and power density of 62.96 W h kg^−1^ and 566.66 W kg^−1^ respectively, as shown in the Ragone plot. A nanofibrous membrane was electrospun and effectively used as a separator in the supercapacitor. Four supercapacitors were assembled in series to demonstrate the device performance by lighting a 2.2 V LED.

Given the current energy crisis and depletion of finite sources, high performance energy storage systems (ESSs), capable of storing energy from both renewable and non-renewable sources, are essential to meet the increasing energy demand. Electrochemical capacitors^1^, also known as supercapacitors, have gained much interest due to their fast charge/discharge rate, long operating life and ability to achieve large specific capacitance^2^. The large specific capacitance is achieved primarily because of two charge storage mechanisms, which occur at the electrode/electrolyte interface. The first is electrical double-layer capacitance (EDLC), which is a non-Faradaic process, and typically uses high specific surface area (SSA) graphitic carbons such as 2-D graphene and 1-D carbon nanotubes (CNT)^3^,^8^ as the electrode materials in order to achieve high power density. The second is pseudocapacitance, a Faradaic process due to the redox reaction^9^,^10^ of metal oxides or electrically conductive polymers which are used as electrode materials to deliver high energy density. These two mechanisms can occur independently or simultaneously, depending on the type of electrode materials used to fabricate the supercapacitor^9^.

Performance of the electrode can be further improved, without compromising the structural integrity and the operating life of supercapacitors, by fabricating hybrid nanocomposites^11^,^12^,^13^,^14^ that uses the two charge storage mechanisms simultaneously. When graphitic carbons are embedded within pseudocapacitive materials such as conducting polymers and metal oxides, hybrid electrodes^15^,^16^,^17^,^18^ are formed which usually achieve superior supercapacitor performance as compared to traditional electrodes. Amongst many graphitic carbon structures, graphene and carbon nanotubes have recently gained much attention due to their extremely large specific surface area^19^,^20^,^21^, a result of atomically thin carbon (2D) structure and extremely high electrical conductivity and superior mechanical properties^22^,^23^,^24^,^25^,^26^,^27^,^28^. Graphene and CNT have been used as an active material for electrodes in supercapacitors exhibiting superior performance in regards to specific capacitance; for example, laser scribed graphene^5^ gave approximately 202 F/g, while bioinspired solvated graphene^29^ based supercapacitors gave about 215 F/g and chemically modified graphene gave about 135 F/g^30^. On the other hand, amongst many pseudocapacitive materials such as conducting polymers like polypyrrole (PPy), polyalinine (PANI) and poly (3-methyl thiophene) (pMeT), PPy has been thoroughly studied and found to possess superior redox performance properties, including low cost, environmental stability and large scale processability^31^,^32^,^33^.

Although nanocomposite electrodes exhibit high specific capacitance, they are limited by their low energy/power densities and high discharge rate. One way to improve the performance of these films lies in the fabrication technique. Several methods have been proposed to fabricate hybrid thin films such as layer-by-layer (LBL) electrostatic self-assembly and spin-coating homogeneously mixed solutions on a substrate^34^,^35^,^36^. However, these fabrication techniques result in agglomerated layers of graphitic materials in the electrode film, leading to poor control over the structural formation and consequently the materials property of the film. In this context, it is critical to tailor the properties of the electrode film by controlling their composition and architecture over its synthesis at a nanometer scale.

In this study, we report a hybridized electrode utilizing EDLC materials such as graphene and CNT within polypyrrole, a pseudocapacitive material, forming a stable and high performance freestanding electrode. This was achieved by electropolymerization of the monomer Py in the presence of graphene and CNT, yielding a nanocomposite polypyrrole electrode. Several nanocomposite electrodes were prepared with increasing concentration of graphene and CNT, such as 0.01 wt%, 0.05 wt % and 0.1 wt% in the *in-situ* polymerization of PPy. Supercapacitor performance depends also on electrochemical window provided by different types of electrolytes, such as aqueous, organic, solvent free ionic liquids or solid state gel electrolytes. It is important to compare the performance of nanocomposite electrodes in various electrolytes. In this study, we analyzed the nanocomposite in three different aqueous electrolytes, namely, ascorbic acid (Vitamin C), sodium sulfate, and sulfuric acid. The highest specific capacitance was observed in the electrode containing graphene and CNT in presence of sulfuric acid electrolyte. Furthermore, while fabricating the supercapacitor device, a novel nanostructured electrospun fiber membrane, made from polycaprolactone (PCL), was used as a separator. Lastly, the performance of this electrode based supercapacitor was measured using a 2.2 V light emitting diode (LED), revealing a slow discharge rate when compared to conventional commercial capacitor. After charging it for 30 s, the LED sustained a light for 3 minutes using a stack of 4 devices in series. The hybrid nanocomposite electrode polypyrrole-CNT-graphene (PCG) will be termed as PCG-*n*, where *n* is the % concentration of the graphene and CNT combined in PPy, henceforth.

## Results

### Fabrication of freestanding nanocomposite electrode

**Figure 1:**
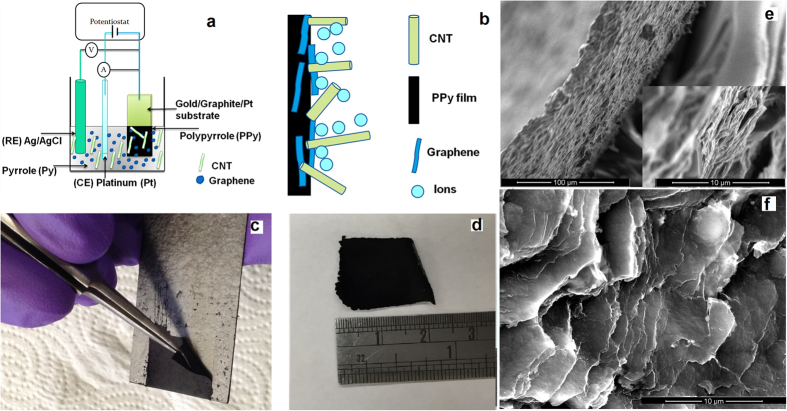
Fabrication and characterization of hybrid electrode. (**a**) Schematic representing the fabrication process of the nanocomposite electrodes. (**b**) Illustration of hybrid nanocomposite film forming a unique interface where graphene and CNT are embedded *in-situ* during polymerization of PPy. (**c**,**d**) Optical image of the actual freestanding film on the graphite substrate with ~2 cm × 2 cm area. (**e**) Cross-sectional SEM micrograph showing layered formation of the polypyrrole film. (**f**) Layers of graphene-CNT coated with PPy during polymerization forming a nanocomposite PCG film.

A hybrid PPy-graphene-CNT freestanding electrode was fabricated using potentiostatic electrochemical polymerization, as shown in Fig. 1(a,b). Schematic illustrates the process by which graphene and CNT are embedded simultaneously while Py is being synthesized, forming a nanocomposite electrode film. As the pyrrole is polymerized, anions are incorporated from the reaction solution, maintaining the charge neutrality in the polymer matrix^37^. This long chain of polypyrrole gets deposited on the negatively charged graphite substrate. The surface of graphite electrode was masked with an insulating scotch tape, exposing an area of 2 cm × 2 cm where the polymer gets deposited. As shown in Fig. 1(c,d), a square film can be easily peeled off after 100 cycles, producing a highly stable conductive electrode with an approximate dimension of 2 cm × 2 cm in area.

As evident from the SEM images (Fig. 1e,f), the control PPy film polymerizes uniformly throughout the process and a typical “cauliflower” like structure can be observed. The average individual grain size of PPy was found to be approximately 6 μm in diameter (Figure S1) and the average distance between two individual neighboring grains is ~300 nm, creating a highly porous electrode. A cross-sectional image reveals that several layers of the PPy film have been synthesized per cycle during polymerization. The overall thickness of the synthesized PPy film is ~75 μm and the average separation between the individual layers of PPy is 150 nm (inset Fig. 1e). This implies that the electrolytic ions are not only accessible from the surface of the electrode film but also from the sides as well, creating a high specific surface area for ion adsorption. In contrast, the PCG 0.01 electrode formed a layered structure, with few stacks of graphene and CNT coated with PPy as shown in Fig. 1f. The approximate thickness of PCG electrode was 30 μm, which was smaller than the PPy electrode. As a result, the PCG film is thinner and displays better electrochemical performance than the PPy film. The average grain size in the PCG electrode was ~6 μm and the distance between two individual grains in PCG is ~650 nm, higher than the control PPy, making it a highly porous structure. The PCG electrodes exhibit more distinct layers than the PPy electrodes, possibly leading to a better electrochemical performance of the PCG electrodes.

**Figure 2:**
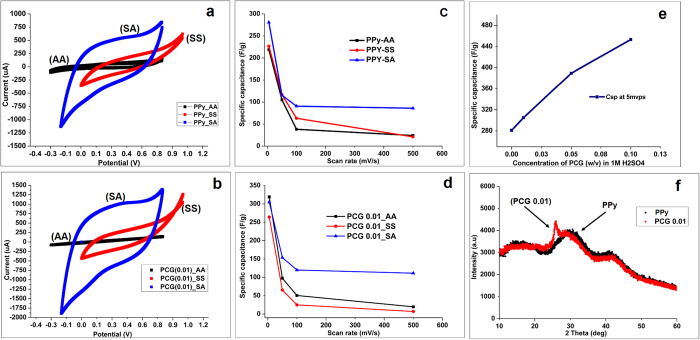
Electrochemical analysis. Cyclic voltammogram of nanocomposites in the presence of three electrolytes (AA, SS and SA). (**a**) Control PPy and (**b**) PCG nanocomposite with 0.01 wt% concentration of graphene and CNT in PPy. (**c**) Specific capacitance of control PPy w.r.t scan rate. (**d**) Specific capacitance of PCG 0.01 at different scan rate. **e**) Specific capacitance with increase in the concentration of graphene and CNT in the PPy. (**f**) X-ray diffraction (XRD) patterns of PPy and PCG nanocomposite.

The effect of crystal formation with and without the presence of graphene and CNT can be observed with the XRD patterns. Figure 2f reveals a formation of a broad peak observed at 2θ = 29.50^o^, which is a characteristic peak of amorphous PPy, and arises mainly due to the scattering from PPy chains at the interplanar spacing. In the case of the PCG 0.01 nanocomposite a sharp peak is observed at approximately 2θ = 25.75^o^ suggesting the incorporation of graphene and CNT in PPy matrix, improving the crystallinity of the structure. Using Bragg’s law, the distance between crystals can be calculated and it is found that the average d_spacing of PPy is 3.024 Å and that of PCG is 3.456 Å. The characteristic peak of the PCG nanocomposite has shifted due to the intercalation of the graphene and CNT in the PPy matrix, resulting in an increase in d_spacing.

### Electrochemical performance of the nanocomposite electrodes

Cyclic voltammetry (CV) tests were performed to identify the charge storage mechanism of the control PPy and hybrid PCG electrodes in three different electrolytes, namely sulfuric acid (SA), sodium sulfate (SS) and ascorbic acid (AA). As shown in Fig. 2(a,b), for both PCG and PPy electrodes, a superior electrochemical performance was observed in the presence of the SA electrolyte. Furthermore, in comparison to PPy, the PCG electrodes always exhibited superior electrochemical activity, as represented by the wider area under the CV curve. Two distinct redox peaks were observed in PCG electrodes in SA, indicating a pseudocapacitive behavior of the PCG electrode. Simultaneously, a pure electrostatic reversible charge storage mechanism was also noted due to the presence of a near rectangular CV curve of the electrodes. Hence, these electrodes store charges via both ion adsorption/desorption and surface redox reactions, making them hybrid in nature.

Based on the CV tests, the specific capacitance was calculated with respect to various scan rates as shown in Fig. 2(c,d). The highest specific capacitance was observed at a scan rate of 5 mV/s in all of the electrode samples. As compared to other electrolytes, the specific capacitance was always higher in the presence of SA, regardless of the samples or scan rates. The PPy electrode in the presence of SA electrolyte yielded the highest specific capacitance of 281 F g^−1^ at 5 mV s^−1^, in comparison to PPy electrodes in SS and AA electrolytes. Along with the highest specific capacitance, the SA electrolyte also exhibited a low decrease in the specific capacitance with a corresponding increase in the scan rate as shown in Fig. 2c. Similarly, the PCG 0.01 nanocomposite exhibits the highest amount of specific capacitance of 305 F/g at a scan rate of 5 mV / s in the presence of SA, which was higher than that of the PPy (Fig. 2d). Interestingly for both PPy and PCG electrodes, after the scan rate of 100 mV s^−1^ the specific capacitance saturated to ~100 F g^−1^ even with a corresponding increase in the scan rate. Furthermore, Fig. 2e demonstrates the effect of an increase in the concentration of graphene and CNT in the overall PPy matrix on its charge storing properties. There was a linear increase in the specific capacitance of the electrodes along with increase in graphene and CNT concentration in pyrrole monomer, achieving the highest specific capacitance of 453 F g^−1^ with the PCG 0.1 electrode at scan rate of 5 mV/s.

### Supercapacitor device assembly and fabrication of nanofibrous separator

**Figure 3:**
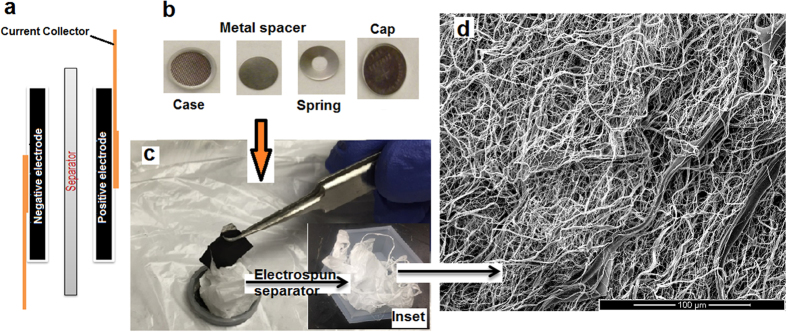
Supercapacitor device fabrication. (**a**) Schematic of the entire device fabrication and its assembly. (**b,c**) Assembly of actual supercapacitor device using CR 2302 coin cell. (**c**) **(inset)** Separator fabricated using electrospinning from polycaprolactone (PCL) nanofibers. (**d**) SEM micrograph of randomly oriented PCL nanofibers.

A supercapacitor device was fabricated as shown in Fig. 3(a–c) where two identical nanocomposite electrodes were placed beside the nanofibrous membrane made from polycaprolactone (PCL) using an electrospinning process (Figure S2). Approximately 3 mL of 1 M sulfuric acid was used as an electrolyte to soak the electrodes in the device. A cathode and an anode case were used from a CR-2032 (MTI corp., USA) type button cell configuration and assembled together as shown in Fig. 3(b,c). SEM micrograph of the electrospun nanofibers is shown in (Fig. 3d) which is used as a separator, having an average fiber diameter of 600 nm. These randomly entangled nanostructres with miniscule fiber diameters and extremely high porosity create a highly permeable membrane which not only acts as electrolyte reserviour but also enables very effeicent ionic kinetics across the membrane, thereby reducing the overall solution resistance and increasing the charge storage mechanism.

**Figure 4:**
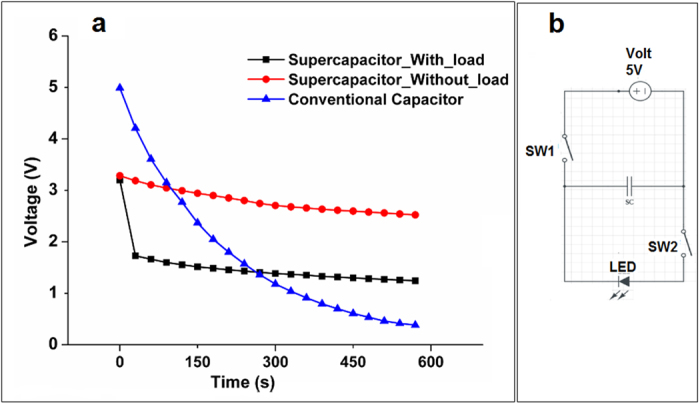
Supercapacitor device testing. (**a**) Discharge profile of the supercapacitor device with and without the presence of an external load (2.2 V LED) with respect to time (min), in comparison with a conventional capacitor. (**b**) Electrical schematic of the implemented circuit to test supercapacitor device.

To further demonstrate the performance of the supercapacitor device, a circuit was implemented to charge and discharge the supercapacitor to an external load, in this case a 2.2 V light emitting diode (LED) was used. In order to light up the LED, a stack of 4 supercapacitor devices were assembled in series and they were enclosed tightly within a CR-2032 button cell holder. The circuit was implemented as shown in Fig. 4b using two switches; SW1 connects the external power supply to the supercapacitor stack while SW2 connects the supercapacitor to the LED. A systematic test case was built to check the performance of the supercapacitor when connected in circuit. When the SW1 is ON and SW2 OFF, the supercapacitor gets charged for 30 s with a constant 5V/3A rating. Upon switching SW1 OFF and SW2 ON, the charged supercapacitors discharges to the LED emitting light, which stays on for a period of 3 min. It was observed that the supercapacitor stack, once charged for about 30 s with 5 V, would achieve an operating voltage up to ~3.25 V and light up the LED for 3 min (supplementary video), indicating high power and energy density of these nanostructured electrodes, along with a capability to store the charges for a longer time. The discharge profile of the supercapacitor stack was plotted in the presence and absence of the LED with time (min) as shown in Fig. 4a. A self-discharge of conventional capacitor (16 μF, 400 V) is also shown for comparison with the fabricated supercapacitor.

## Discussion

The novelty of the electrode fabrication technique lies in the ease of the *in-situ* synthesis of polypyrrole-CNT-graphene nanocomposite resulting in a controlled layered structure with high porosity. The resulting electrode has high ion accessibility, due to the fact that no organic binders were used to fabricate the thin film, which would otherwise reduce the ionic access to the active materials in the supercapacitor electrodes. This fabrication technique is also useful to develop micro-supercapacitors and biosensors because of its ability to precisely deposit the conductive nanocomposite material on the existing circuits.

**Figure 5:**
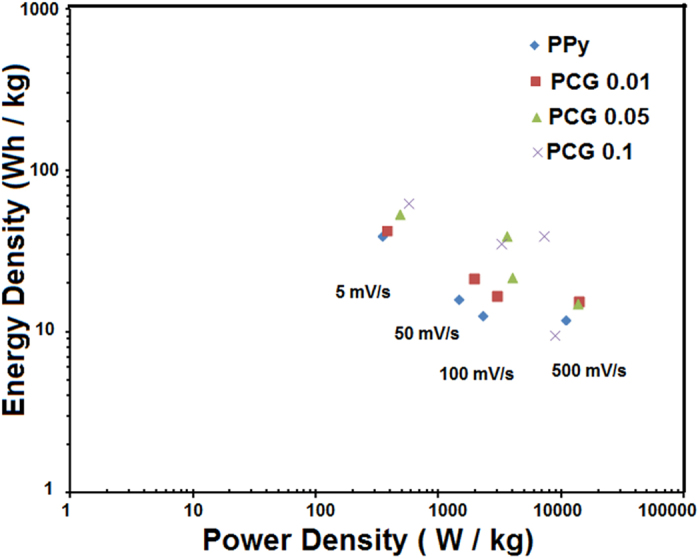
Ragone plot of nanocomposite electrodes. Energy and power density calculated of varying concentrations of graphene and CNT at different scan rates (5–500 mV/s).

The electrochemical performance of the nanocomposite electrode was improved by increasing the concentration of graphene and CNT when incorporated in PPy as shown in the Ragone plot in Fig. 5. The highest concentration of PCG nanocomposite (0.1 wt %) resulted in the highest energy density value of 62.96 W h kg^−1^ and a power density of 566.66 W kg^−1^ at the scan rate of 5 mV/s. For the PCG 0.1, an ultrahigh energy density was obtained in the range of 9.62 W h kg^−1^–62.96 W h kg^−1^ and a power density in the range of 1416.66 W kg^−1^–8665 W kg^−1^ at various scan rates. These electrodes exhibit high power density from EDLC materials and simultaneously exhibit high energy density from the pseudocapacitive type of materials which could results in many high power applications. The power density (P, in W kg^−1^) and the energy density (E, W h kg^−1^) of the electrode material was calculated by using the following expressions
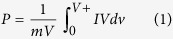


Here, V is the initial voltage during discharge; m is the mass of electrodes
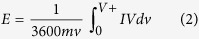


Here *v* is the scan rate (Vs^−1^)

The use of electrospun nanofibers as a separating membrane has also improved the performance of the supercapacitor device. Owing to small fiber diameter (~600 nm) and high surface area, the fibrous nanostructures act not only as an efficient permeable membrane across the hybrid electrodes but also as an electrolytic reservoir, which helps to slow the overall discharge in device. The supercapacitors can be integrated into many applications such as microelectrode arrays^38^,^39^, and in microelectronics because of their scalability and ease of fabrication along with low cost. They could also be used on flexible substrates such as fabrics, roll-up portable and flexible display^40^,^41^.

## Methods

### Fabrication of nanocomposite electrode films

The polypyrrole (PPy) film was prepared using pyrrole (Py) (Sigma Aldrich, USA) monomer solution. The Py solution was prepared by stirring 0.87 ml of Py along with 0.355 g of Na_2_SO_4_ as supporting electrolyte in 25 mL of DH_2_O for 1 h. To make PCG nanocomposite with concentrations 0.01% w/v (PCG0.01), 0.05% w/v (PCG0.05) and 0.1% w/v (PCG0.1), an equal amount of graphene and CNT (1.25 mg, 6.25 mg and 12.5 mg, respectively) were dispersed in 25 ml Py solution. This solution was stirred magnetically for 1 h, followed by sonication for 2 h to ensure that graphene and CNT were dispersed thoroughly. PPy and PCG films were synthesized potentiostatically at the scan rate of 20 mV s^−1^ and 10 mV s^−1^ respectively. The potential window for both samples was from [800–900 mV] and they were scanned for 100 cycles to obtain free standing films.

### Aqueous electrolyte preparation

The performance of electrodes was studied in the presence of different electrolytes such as ascorbic acid (AA), sodium sulfate (SS) and sulfuric acid (SA). AA electrolyte was prepared using 10 mM Ascorbic Acid in 50 mM Phosphate Buffer Saline (PBS). 0.5 M of SS electrolyte was prepared by adding weighted amount of sodium sulfate salt to deionized water and stirring until a clear solution was achieved. As SS is a neutral salt, its pH is near 7. Lastly, sulfuric acid, which was the strongest electrolyte used, had a pH close to 0 in a 1 M solution.

### Electrochemical analysis

The nanocomposite electrodes were characterized electrochemically (eDaq, Model number ER466) using cyclic voltammetry. A three-electrode setup, consisting of a Pt counter electrode, an Ag/AgCl reference electrode and a PPy or PCG-n as working electrode was used for the electrochemical analysis. The capacitance values were calculated from the cyclic voltammogram using the following equation
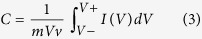


Where *C* is the specific capacitance (F g^−1^), *m* is the mass of the active electrode material (g), [*V = V*_*+*_*−V*_*−*_] is the potential difference and *v* is the scan rate (m V s^−1^). The total charge transferred during the cycle can be calculated by integrating the area under the curve of CV. The potential difference for each electrolyte was, V_AA_ [−300 mV–800 mV], V_SS_ [0–1,000 mV], and V_SA_ [−200–800 mV]. The electrodes were weighed before and after the deposition on graphite electrode in order to calculate the mass of the active nanocomposite material. A 3 mm cylindrical graphite rod was used for all experiments and it was covered using an insulating tape on its surface leaving only the base uncovered for deposition. This ensured that same surface area was maintained during each experiment.

### Materials characterization

The structural morphology of the nanocomposite films were characterized using scanning electron microscope (SEM, FEI Quanta 250 FEG). X-ray diffraction study was performed using Bruker Advanced diffractometer. The XRD patterns were recorded in the 2θ range of 10^o^–80^o^ using CuKα radiation (λ = 1.5406 Å) operated at 40 kV and 40 mA. Using Bragg’s law, the distance between crystals were calculated as follows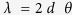 , here λ is the X-ray wavelength and θ is the diffraction angle at the maximum intensity and d is the average spacing between the planes in the atomic lattice.

## Additional Information

**How to cite this article**: Aphale, A. *et al*. Hybrid Electrodes by *In-Situ* Integration of Graphene and Carbon-Nanotubes in Polypyrrole for Supercapacitors. *Sci. Rep*. **5**, 14445; doi: 10.1038/srep14445 (2015).

## Supplementary Material

Supplementary Information
