# Supplementary material for: Hybrid Electrodes by In-Situ Integration of Graphene and Carbon-Nanotubes in Polypyrrole for Supercapacitors
Source: Sci Rep. 2015 Sep 23;5:14445. doi: 10.1038/srep14445 (PMC4585780; doi:10.1038/srep14445)
Supplement: Supplementary Information [file srep14445-s1.pdf]

Supplementary Information

Hybrid Electrodes by *In-Situ* Integration of Graphene and Carbon-Nanotubes in Polypyrrole for Supercapacitors

Ashish Aphale[1], Krushangi Maisuria[2], Manoj Mahapatra[3], Angela Santiago[4], Prabhakar Singh[3] and Prabir Patra[1, 5 *]

[1] Department of Biomedical Engineering, University of Bridgeport, CT-06604

[2] Fairfield Ludlowe High School, Fairfield, CT-06824

[3] Department of Materials Science and Engineering, Center for Clean Energy Engineering, University of Connecticut, Storrs, CT-06269

[4] Department of Chemistry, University of Bridgeport, CT-06604

[5] Department of Mechanical Engineering, University of Bridgeport, CT-06604

*Corresponding author: Professor Prabir K. Patra
Department of Biomedical Engineering and Department of Mechanical Engineering,
School of Engineering, University of Bridgeport, 126 Park Ave, Bridgeport, CT-06604 USA
Email: ppatra@bridgeport.edu
Ph: 1-203-576-4165
Fax: 1-203-576-4343

KEYWORDS: supercapacitor, conductive polymers, cyclic voltammetry, graphene, carbon nanotubes

Scanning electron microscopy (SEM) of the nanocomposite samples

Figure S1 A is a SEM of the control graphene with the approximate flake size of 300 nm. The average individual grain size of PPy is approximately 6 μm in diameter and the average distance between two individual neighboring grains is ~300 nm (Figure S1 B, C). Meanwhile, in Figure S1 D, the approximate grain size in a PCG nanocomposite was also ~ 6 μm and the distance between two individual grains in PCG was ~ 650 nm (Figure S1 D) making the PCG nanocomposite is more porous than the PPy nanocomposite. A cross-sectional SEM micrograph of the PCG film (Figure S1 F) reveals the width of the film to be 30 μm.

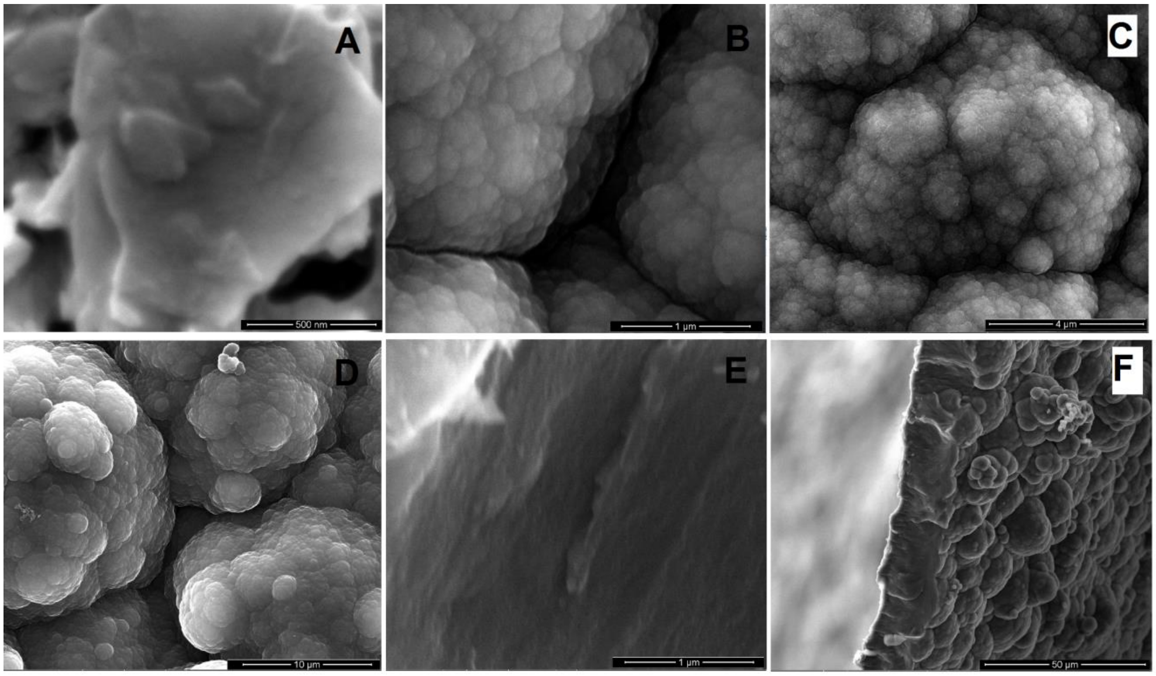

Figure S1: Morphological characterizations. A) Micrograph of control graphene, B & C) micrograph of control PPy film, D & E) micrograph of the polypyrrole- graphene-CNT (PCG) nanocomposite, F) Cross-sectional SEM of the PCG nanocomposite.

Fabrication of PCL based highly porous permeable membrane

Electrospinning was setup with a dual syringe pump (KDS 200 series, KDS scientific), a high voltage power supply (Model ES30P, Gamma High Voltage Research) and a metallic (aluminum) collector placed at an appropriate distance from the tip of the needle. The variables that are important in electrospinning of nanofibers are the distance between the needle and the collector (*d*), viscosity of the polymer solution, flow rate (*r*) of the polymer solution from the needle tip through the syringe and electric field strength applied which is voltage per distance (kV/cm) between the needle and the collector. 10 wt% PCL solutions (Sigma Aldrich, 70k-90k Mw) was prepared using 10g of PCL pallets in 100ml acetone solvent and stirring at 60°C until a clear solution was achieved. Polymeric nanocomposite solution was also prepared by adding different concentrations of graphene in 10 wt% PCL solutions[1].

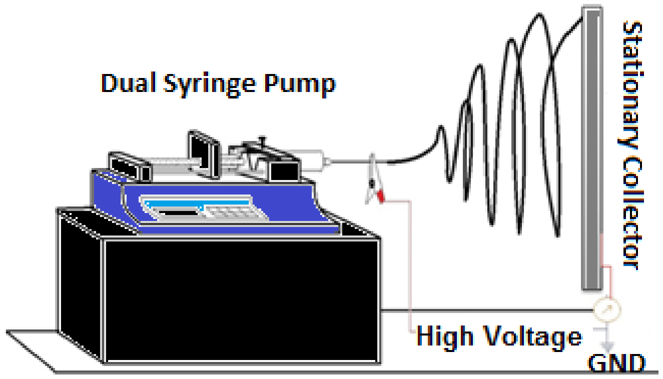

Figure S2: Electrospinning setup for the fabrication of PCL nanofibers as a separator of supercapacitor

Reference:

1. Aphale, A. N., Mahakalkar, K., Macwan, I. G., Mukerji, I., Cox, P., Mahapatra, M., Singh, P., Ajayan, P. M. & Patra, P. K. Fabrication and Experimental Analysis of Axially Oriented Nanofibers. *J. Nanosci. Nanotechnol.* **15,** (2015) (In Press).
